# Structural Insights into the Assembly of CARMA1 and BCL10

**DOI:** 10.1371/journal.pone.0042775

**Published:** 2012-08-03

**Authors:** Siwei Li, Xue Yang, Juan Shao, Yuequan Shen

**Affiliations:** 1 State Key Laboratory of Medicinal Chemical Biology, Nankai University, Tianjin, China; 2 College of Life Sciences, Nankai University, Tianjin, China; University of Washington, United States of America

## Abstract

The CBM complex (CARMA1, BCL10 and MALT1) plays a crucial role in B and T lymphocyte activation. CARMA1 serves as a scaffold for BCL10, MALT1 and other effector proteins and regulates various signaling pathways related to the immune response. The assembly of CARMA1 and BCL10 is mediated through a CARD-CARD interaction. Here, we report the crystal structure of the CARD domain of CARMA1 at a resolution of 1.75 Å. The structure consists of six helices, as previously determined for CARD domains. Structural and computational analysis identified the binding interface between CARMA1-CARD and BCL10-CARD, which consists of a basic patch in CARMA1 and an acidic patch in BCL10. Site-directed mutagenesis, co-immunoprecipitation and an NF-κB activation assay confirmed that the interface is necessary for association and downstream signaling. Our studies provide molecular insight into the assembly of CARMA1 and BCL10.

## Introduction

Lymphocyte activation is emerging as a critical process in the immune and inflammatory responses. The occurrence of infection, immune disease and tumors are all tightly related to the dysregulation of lymphocyte activation. Stimulated lymphocyte cell-surface antigen receptors (AgR) in association with co-stimulatory receptors trigger a series of signal transduction pathways that lead to the activation and proliferation of lymphocytes [1]. The recognition of the T-cell receptor (TCR)/CD3 complex by peptide-major histocompatibility complexes on antigen presenting cells (APC) in concert with the co-stimulation of CD28 leads to the activation of cytosolic Src and Syk family tyrosine kinases. These stimulated tyrosine kinases and various adapter proteins further recruit other effector proteins [2], including protein kinases and phosphatases, which are implicated in signal transduction pathways that lead to the activation and proliferation of T cells. Alternatively, B cell activation is similar to T cell activation; however, the initiation of the stimulated signal depends on the B-cell receptor (BCR). The signals are triggered by lymphocyte surface receptors and further transduced by a multitude of effectors that converge on the CBM [3], which is a macromolecule complex that consists of caspase recruitment domain membrane-associated guanylate kinase protein 1 (CARMA1), B-cell lymphoma 10 (BCL10) [4] and mucosa-associated lymphoid tissue lymphoma translocation gene 1 (MALT1) [5]. The CBM complex plays a specific and essential role in the activation of nuclear factor-κB (NF-κB) and c-Jun N-terminal kinase (JNK) [6], [7], [8]. The mutation or deficiency of the components of the CBM complex impairs lymphocyte proliferation and activation, specifically activation induced by NF-κB [3], [9], [10]. NF-κB is a key transcription factor activated by AgR-dependent cascades, which regulates the expression of various genes involved in immunity [11]. In resting cells, NF-κB interacts with IκB, which inhibits the function of NF-κB by sequestering NF-κB in the cytoplasm. Upon stimulation with various antigens and cytokines, IκB proteins are phosphorylated and undergo rapid ubiquitination-dependent degradation, which requires the IκB kinase (IKK) complex, and leads to NF-κB translocation into the nucleus where it regulates gene transcription [12]. The CBM complex plays an essential role in the activation of IKK [11], triggers NF-κB activation and participates in the regulation of the immune response. While defective IKK activation appears in CARMA1-/- lymphocytes [13], several oncogenic mutations of CARMA1 have been found in patients with diffuse large B-cell lymphoma [14]. Furthermore, lymphomas of mucosa-associated lymphoid tissue result from chromosomal translocation of genes encoding MALT1 [15] or aberrant expression of BCL10 [16].

CARMA1 (also named CARD11) [17] contains a caspase-recruitment domain (CARD), a coiled-coil (CC) domain at the N-terminus and a membrane-associated kinase (MAGUK) domain at the C-terminus. It belongs to the MAGUK family, which is a class of proteins with C-terminal PDZ-SH3-GUK tripartite modular domains. B-cell lymphoma 10 (BCL10) is composed of a CARD domain and a Ser/Thr-rich C-terminal domain of unknown structure, which is highly phosphorylated in response to stimulation [18]. As an adapter molecule, BCL10 interacts with CARMA1 through their CARD motifs and forms a complex with MALT1 [19]. MALT1 consists of an N-terminal death domain followed by two Ig-like domains, a C-terminal caspase-like domain and another Ig-like domain. The two successive Ig-like domains are correlated in the interaction with BCL10, which requires the BCL10-CARD and a short sequence of amino acids adjacent to the CARD domain [20].

As a scaffold molecule, CARMA1 recruits BCL10, MALT1, protein kinase C and TRAF6 to form a multiprotein complex. The dynamic assembly of the CBM complex under stimulation with AgR transmits the upstream signals to IKK and ultimately activates NF-κB [17], [21]. In vivo studies, the reconstitution of CARMA1 fully rescued the signaling defect of CARMA1-deficient cells, and overexpression of CARMA1 resulted in the activation of NF-κB. The CC domain of CARMA1 is hypothesized to mediate oligomerization and association with the preformed BCL10-MALT1 complex, which appears to be constitutively present in the cytoplasm [22] and contributes to the formation of the CBM complex. The function of the MAGUK domain remains unclear; however, mutation studies suggest that the SH3 domain regulates the membrane localization of CARMA1 [23].

It is hypothesized that inactive CARMA1 exists in an autorepressed conformation [9], [24]. The linker region between the CC and MAGUK domains block the homotypic interaction of the CARD motifs by binding to the N-terminus of CARMA1, which interferes with the formation of the CBM complex. A series of phosphorylation events that occur in the linker region are mediated by PKC, IKKβ and HPK, which contribute to a conformational change in CARMA1 [25], [26]. The activated conformation allows for the combination of CARD domains, which induces the assembly of the CBM complex.

The CARD domains mediate protein-protein interactions that belong to the caspase-recruitment domain subfamily of the death domain superfamily [27], [28]. The members of this subfamily are characterized by their conserved structures that contain six helices. All CARD domains all posses an acidic and basic patch on different sides. The interaction of CARD domains requires amino acids with opposite charges; for example, the basic surface of the Apaf-1 CARD interacts with the acidic surface of the procaspase-9 CARD [29]. Compared to available CARD domain structures, the CARD domains of CARMA1 and BCL10 may interact with each other using a similar mechanism. Mice with CARMA1 lacking the CARD domain exhibit defective B lymphocyte development and impaired proliferation of B and T lymphocytes [30].

To further characterize the molecular basis of the interaction of CARMA1 and BCL10, we have determined the crystal structure of the CARD domain from CARMA1 (CARMA1-CARD). Furthermore, we have identified several critical residues that contribute to the interaction between CARMA1 and BCL10 using site-directed mutagenesis, co-immunoprecipitation and an NF-κB luciferase assay. Our study provides a molecular basis for the assembly of CARMA1 and BCL10.

## Results

### Structure of CARMA1-CARD

**Figure 1:**
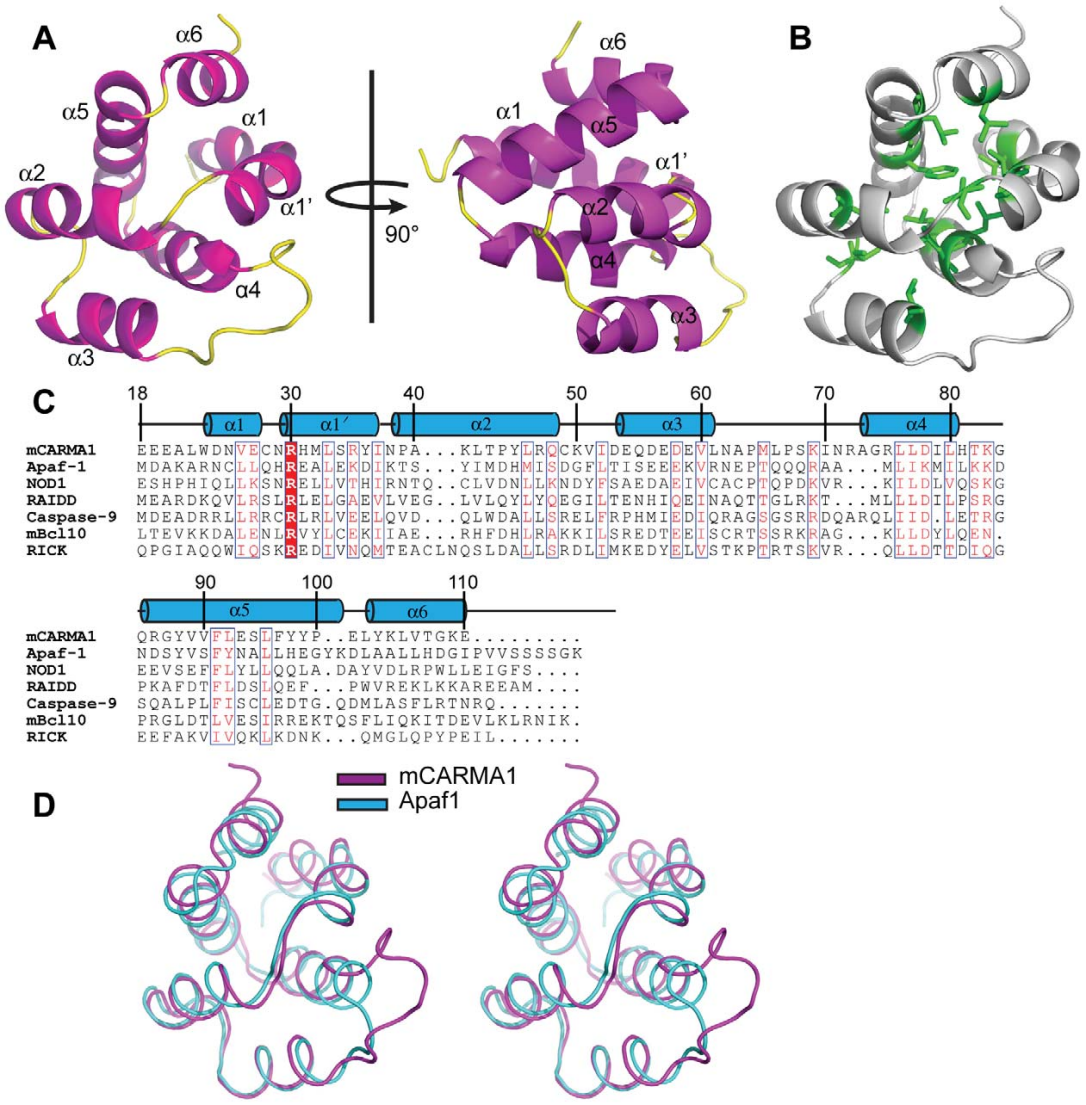
Crystal structure of CARMA1-CARD and comparison with other CARD family members. (**A**) Cartoon representation of CARMA1-CARD. The six helices are colored magenta, and the loops are colored yellow. (**B**) The conserved central hydrophobic core. The conserved hydrophobic residues are essential for the stabilization of the structure. The side chains are shown as sticks and are colored green. (**C**) Sequence alignment of CARMA1-CARD and seven representative CARD domains. Conserved residues are labeled and colored red. Secondary structures (helices α1 to α6) are shown above the sequence. (**D**) A stereo view of the structural superposition of CARMA1-CARD and Apaf-1-CARD is shown in magenta and cyan, respectively.

The crystal structure of CARMA1-CARD was determined at a resolution of 1.75 Å using single-wavelength anomalous dispersion (SAD). There is one molecule per asymmetric unit. The structure of CARMA1-CARD is presented in Figure 1A. Each molecule contains six α-helices, and they are compactly arranged around a conserved hydrophobic core (Figure 1B), which is the characteristic fold in the death domain superfamily [29]. The structure of CARMA1-CARD contains residues 19–109. The central hydrophobic core consists of residues V26, L33 and I37 from H1; A40 and L46 from H2; I52 and V60 from H3; L76, L77 and L80 from H4; F91 and L95 from H5; and L102 from H6. These residues are conserved among all members of the CARD family (Figure 1C) and serve as a molecular skeleton that stabilizes the conformation of the CARD domain. Helices H2 to H5 are arranged in an anti-parallel helix bundle, whereas H1 and H6 flank one side of the bundle and cover the top region of helices H4 and H5. Of the six helices, H1 is the longest and is slightly bent at residue N29, thereby separating the helix into two smaller α-helices, which is similar to the CARD domains from Apaf-1 and NOD1 [28], [29].

### Comparison with other CARD domains

DALI server search results reveal that several proteins share similar structural features to the CARMA1-CARD, including Apaf1 (Z-score = 14.4, rmsd of 1.5 Å for 90 aligned Cα), Iceberg (Z-score = 11.3, rmsd of 2.0 Å for 89 aligned Cα) and RAIDD (Z-score = 8.0, rmsd of 2.2 Å for 100 aligned Cα). Although sequence homology between CARD domains is low (sequence identity between CARMA1-CARD and Apaf-1, Iceberg and RAIDD is 18.9%, 17.9% and 18.7%, respectively), the structure of CARD domains is highly conserved (Figure 1D). CARD/CARD interactions between RAIDD and caspase2 and Apaf-1 and procaspase-9 play important roles in the apoptosis signaling pathway [27], [29], and the recognition of CARD domains between CARMA1 and the adaptor protein BCL10 is critical for the immune response. Similar to Apaf-1, CARMA1-CARD has similar features in terms of helix length and orientation. The significant difference exists in the loop between helices H3 and H4, which is longer and more flexible in CARMA1-CARD (Figure 1D).

### Positively charged surfaces of the CARMA1-CARD domain

**Figure 2:**
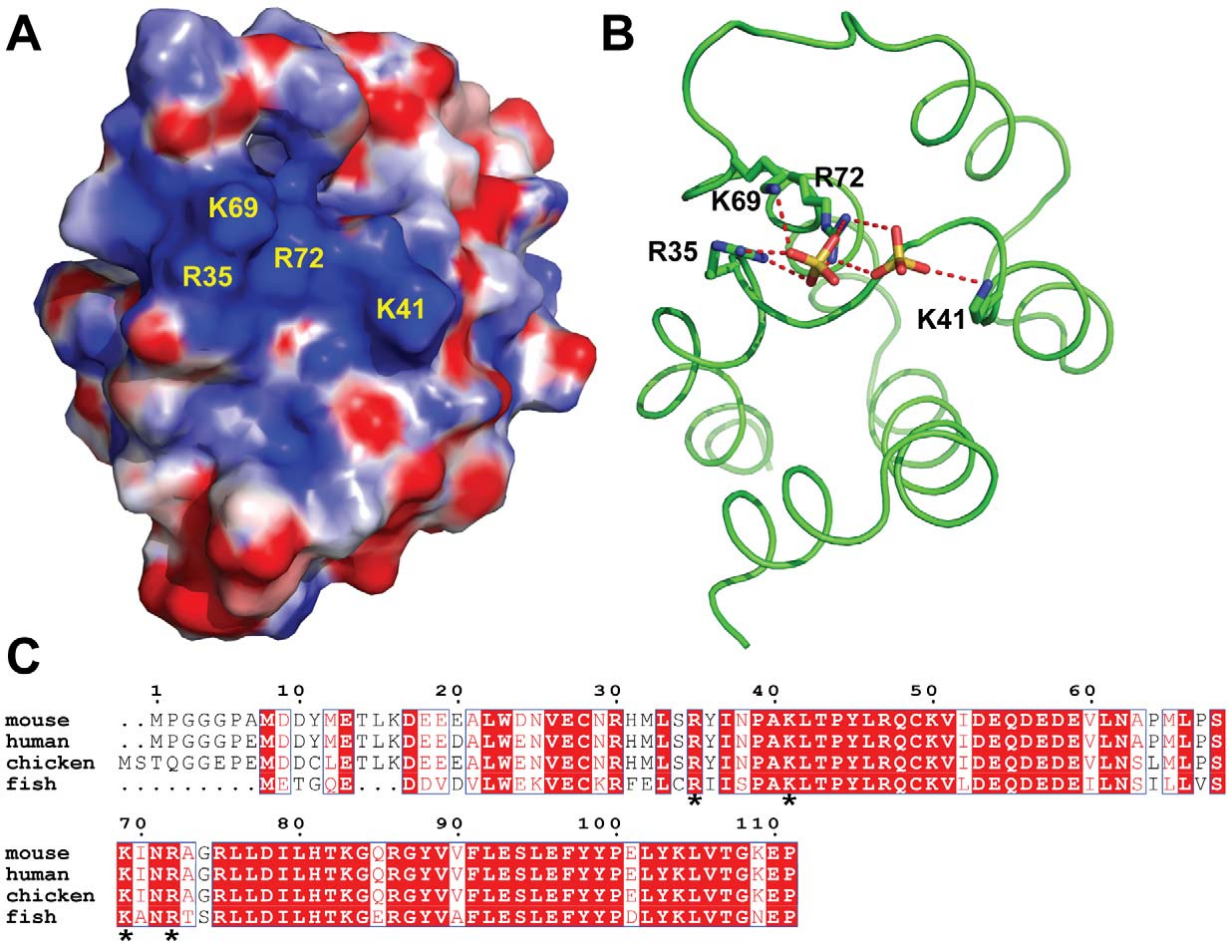
The binding surface of CARMA1-CARD. (**A**) The representative basic residues (R35, K41, K69 and R72) on the positive surface of the CARMA1-CARD are colored blue. (**B**) Interactions between the basic residues and surrounding sulfate ions. The side chains of basic residues R35, K41, K69 and R72 and sulfate ions are shown as sticks. The oxygen atoms and sulfur atoms are colored red and yellow, respectively. Hydrogen bonds are shown as red dashed lines. (**C**) Sequence alignment of CARMA1-CARD proteins from different species. The conserved amino acids are highlighted in red. Conserved residues in the basic patch are denoted with asterisks.

To map the binding site of CARMA1-CARD with BCL10, we investigated the electrostatic surface of the CARMA1-CARD structure. An area of clustered positive charges was found on the surface of CARMA1-CARD (Figure 2A). The positive patch was formed by residues R35, K41, K69 and R72. In the crystal structure of CARMA1-CARD, this positive patch is stabilized by two sulfate ions through multiple hydrogen bonds (Figure 2B). Moreover, sequence alignment between different species reveals that these four residues are highly conserved (Figure 2C). Previous studies of CARD-CARD complexes revealed that the interactions between two CARD domains mainly rely on electrostatic interactions [27], [28], [29]. For example, in the Apaf-1/procapase-9 complex, the acidic surface of Apaf-1 and basic surface of procapase-9 contribute to the formation of the CARD-CARD complex [29]. Therefore, we hypothesize that the positively charged surface of CARMA1-CARD may play an important role in an electrostatic interaction between CARMA1 and BCL10.

### Docking studies of BCL10

**Figure 3:**
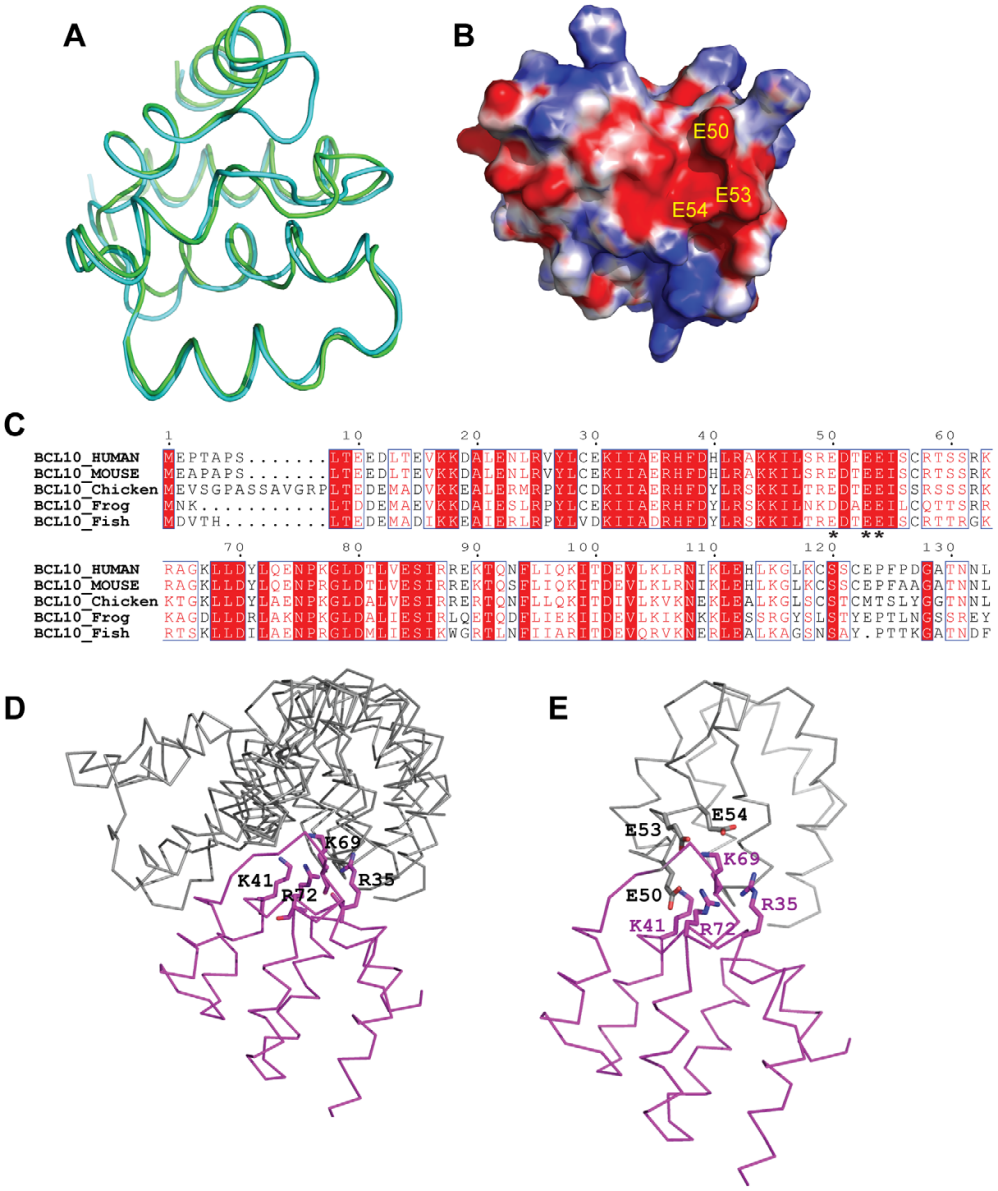
The binding surface of BCL10-CARD. (**A**) Superposition of homology models of BCL10-CARD. The computational results from the programs MODELLER and SWISS-MODEL are represented with green and blue cartoon models, respectively. (**B**) The electrostatic surface of BCL10-CARD. Red: negative; blue: positive; and white: neutral. Residues E50, E53 and E54 are labeled. (**C**) Sequence alignment of BCL10-CARD proteins from different species. The conserved amino acids are highlighted with red, and the conserved acidic residues that make up the acidic patch are denoted with asterisks. (**D**) Protein docking models of the CARMA1-CARD and BCL10-CARD calculated using the ZDOCK server. CARMA1-CARD and BCL10-CARD are colored magenta and gray, respectively. Three out of ten best scoring complexes place the BCL10-CARD domain approaching the interface containing residues R35, K41, K69 and R72 of CARMA1-CARD. The side chains of residues R35, K41, K69 and R72 are shown as sticks. (**E**) The interactions between CARD domains in the best docking complex model. CARMA1-CARD and BCL10-CARD are colored magenta and gray, respectively. Side chains of E50, E53 and E54 of BCL10 as well as R53, K41, K69 and R72 of CARMA1 are shown as stick.

The BCL10-CARD domain is notoriously difficult to purify; therefore, we took advantage of computational methods. Homology models of BCL10-CARD were constructed using MODELLER [31] and SWISS-MODEL software [32]. Both BCL10-CARD models are similar and have an rmsd of 1.4 Å (Figure 3A). As expected, the model of BCL10-CARD shows a negatively charged surface (Figure 3B). The surface is composed of residues E50, E53 and E54, and these residues are highly conserved across different species (Figure 3C). Protein docking of the CARMA1-CARD and BCL10-CARD were used to explore potential binding interfaces [33]. Three best-docked complex models converged the BCL10-CARD around the interface with the positively charged surface in CARMA1-CARD (Figure 3D, E).

### Interactions between CARMA1-CARD and BCL10-CARD

**Figure 4:**
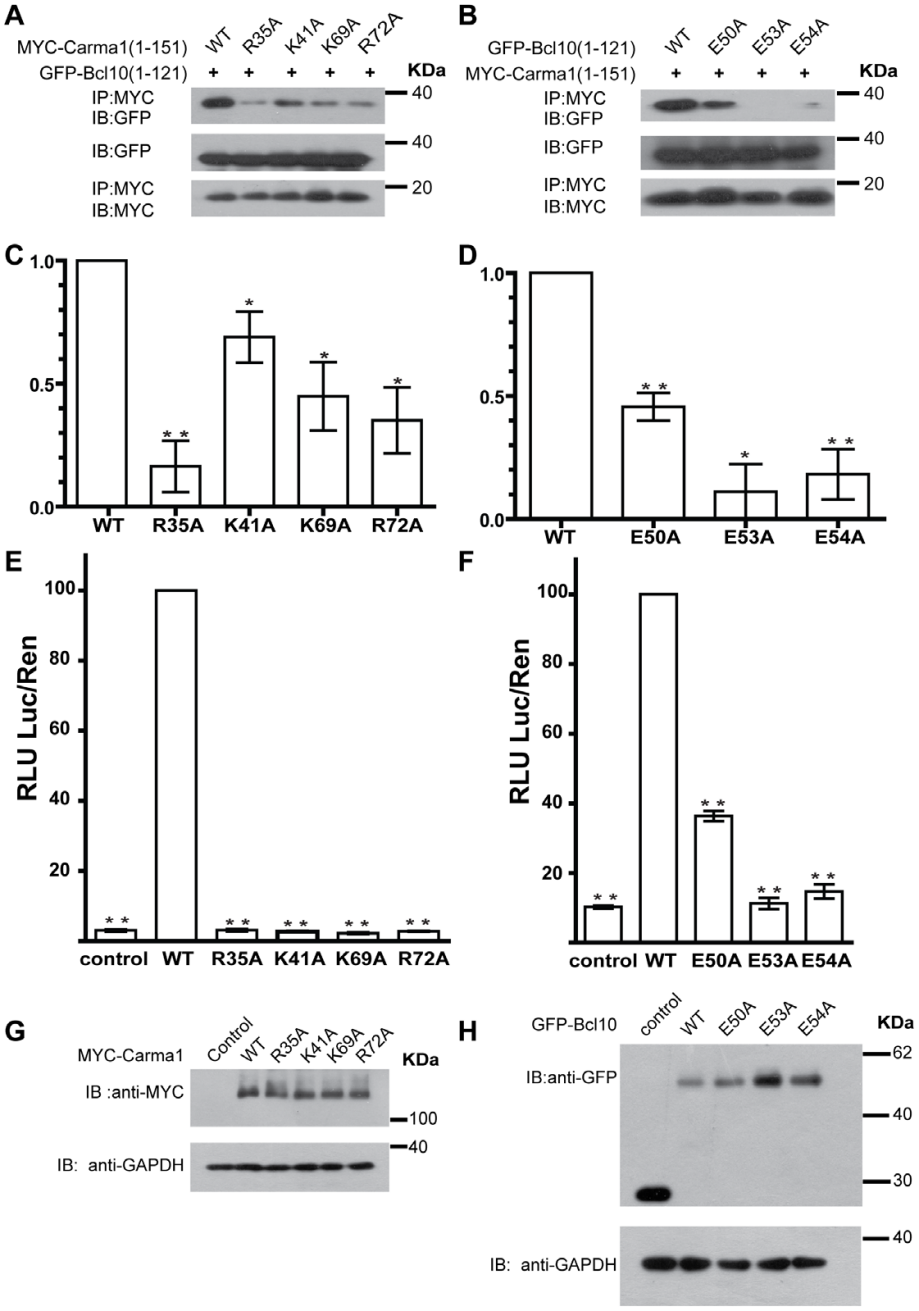
Association of CARMA1 and BCL10. (**A**) Co-IP analysis of interactions between BCL10-CARD and variants of CARMA1-CARD. HEK293T cells were transiently co-transfected with GFP-tagged BCL10-CARD and wild type or mutants of Myc-tagged CARMA1-CARD constructs. Cell extracts were immunoprecipitated using an anti-Myc antibody and blotted using anti-GFP. (**B**) Co-IP analysis of interactions between Myc-tagged CARMA1-CARD and wild type or mutants of GFP-tagged BCL10-CARD constructs. (**C,D**) Bar graph displaying interactions between CARMA1-CARD and BCL10-CARD. (**E**) The effect of wild type and mutants of CARMA1 on the NF-κB reporter assay. (**F**) The effect of wild type and mutants of BCL10 on NF-κB activity. RLU: relative luciferase unit; Luc: firefly luciferase activity; and Ren: Renilla luciferase activity. The error bars indicate the standard error of the mean (n = 3 separate experiments). * indicates a P value<0.05, ** indicates a P value<0.001. (**G, H**) The expression levels of CARMA1 and BCL10 in NF-κB assays were checked by immunoblotting with anti-GFP, anti-MYC and anti-GAPDH antibodys, respectively.

To verify the computational results, we created Myc-fused mutants of the residues (R35A, K41A, K69A and R72A) of the positively charged surface of CARMA1-CARD and GFP-fused mutants of the residues (E50A, E53A and E54A) of the negatively charged surface of BCL10-CARD and performed co-immunoprecipitation assays to observe binding. In comparison to wild type CARMA1-CARD, the R35A mutant exhibited significantly attenuated (less than 20%) binding of CARMA1-CARD to BCL10-CARD, and two mutants (K69A and R72A) exhibited moderately attenuated (less than 50%) binding of the two CARD domains. In contrast, the K41A mutant showed slightly attenuated (less than 80%) interactions between the two CARD domains (Figure 4A, C). Moreover, compared to wild type BCL10-CARD, all three mutants (E50A, E53A and E54A) exhibited decreased binding between the two CARD domains (Figure 4B, D).

To confirm the functional importance of these seven mutations, we generated full-length CARMA1 and BCL10 mutants and performed the NF-κB activation-dependent dual luciferase reporter assay in HEK293T cells. The raw data of the luciferase assay was shown in Table S1. Four mutants (R35A, K41A, K69A and R72A) of CARMA1 all yielded the most severely (less than 10%) attenuated luciferase signals (Figure 4E, G). Similarly, three mutants (E50A, E53A and E54A) of BCL10 exhibited moderately (less than 40%) attenuated luciferase signals (Figure 4F, H). These results indicate that mutation of key residues on the surface of CARMA1 and BCL10 has a negative impact on the association between the proteins and affects downstream signaling.

## Discussion

The CBM signalosome is assembled through a CARD/CARD interaction between CARMA1 and BCL10 and a CC/Paracaspase interaction between CARMA1 and MALT1. The CARD domain is located at the amino terminus of CARMA1 and is able to associate with BCL10 [34], the key first step in CBM formation after stimulation. We determined the crystal structure of the CARMA1-CARD at a high resolution and have provided structural insight into the assembly of CARMA1 and BCL10. In our structure, the CARMA1-CARD shares similar structural features with other CARD domains, including six conserved helices and a positively charged surface. Protein docking of CARMA1 and BCL10, which was based on the CARMA1-CARD structure and homology model of BCL10-CARD, suggests that the basic patch of CARMA1-CARD is coupled with the acidic patch of BCL10 and that both structural features contribute to the formation of the complex. Furthermore, mutation of the main residues of the basic surface (R35, K41, K69 and R72) of the CARMA1-CARD and acidic surface (E50, E53 and E54) of the BCL10-CARD in combination with co-IP assays demonstrated that these residues are involved in the interaction of the two molecules. Subsequent NF-κB reporter assays further confirmed their functional importance.

Previous studies have shown that electrostatic interactions between the charged surface residues mediate a mutual recognition of CARD/CARD domains [27], [28], [29]. However, low sequence homology among CARD family members indicates that their partner recognition and functional specificity may require other factors. In the Apaf-1/procaspase-9 complex, extensive hydrogen bond contacts and van der Waal interactions within the interface, not electrostatic interactions, serve as the dominant force [29]. The NOD1/RICK complex appears to have similar stability as the Apaf-1/procaspase-9 complex at high ionic-strength conditions. Within the interface of the complex, the hydrophobic interactions adjacent to the charged interface further strengthen the electrostatic interactions [28]. In the CARMA1/BCL10 complex, electrostatic interactions predominantly contribute to their association, which appears to be different from other CARD-CARD interactions that have been identified. It is speculative that the CC domain of CARMA1 binding to the Paracaspase domain of Malt1 cooperatively enhances the association of CARMA1 and BCL10 within the CBM complex [35].

The CBM complex is known to regulate several key signaling pathways in the immune system [3]. The structure of CARMA1-CARD sheds light on the molecular mechanism of the assembly of the ternary complex. Future efforts investigating CARMA1 and its partners will uncover the critical mechanisms for lymphocyte regulation.

## Materials and Methods

### Protein expression and purification

The gene encoding residues 18–110 of mouse CARMA1 protein (CARD protein) was cloned into modified pET-32a (Novagen) vector, in which the thrombin site and the S tag were replaced by the Prescission site (Leu-Glu-Val-Leu-Phe-Gln-Gly-Pro) and transformed into *E. coli* BL21-Codon Plus cells. The resulting cells were then grown in LB medium at 37°C to A_600_ = 0.6, induced by isopropyl-1-thiogalactopyranoside (IPTG) to a final concentration of 200 µM at 25°C and harvested overnight.

The cells were lysed in buffer A (20 mM Tris-HCl pH 8.5, 500 mM NaCl and 0.1 mM PMSF) by lysozyme (0.1 mg/ml) and sonication. After centrifugation at 20,000 g for 60 min, the supernatant was loaded onto a Ni-NTA column, washed with buffer B (20 mM Tris-HCl pH 8.5, 500 mM NaCl, 0.1 mM PMSF and 20 mM imidazole) and eluted with buffer C containing 300 mM imidazole. The eluted 6His-tagged protein was digested by Prescission protease at 4°C overnight and further purified by reversal Ni-NTA column and gel filtration chromatography. The protein was concentrated to 15 mg/ml in a buffer containing 20 mM Tris-HCl pH 8.5, 300 mM NaCl and 1 mM dithiothreitol (DTT).

The seleno-methionine (Se-Met) labeled derivative protein were produced following the same protocol as that of the wild-type protein, with the exception that methionine auxotroph *E. coli* B834(DE3) cells [36], [37] and minimal medium were used to express the recombinant protein.

### Crystallization and data collection

The CARD protein (8.4 mg/ml in 20 mM Tris-HCl, pH 8.5, 300 mM NaCl and 1 mM DTT) was crystallized using the sitting drop vapor diffusion method equilibrated against a reservoir solution of 1.6 M MgSO_4_ and 0.1 M MES pH 6.5. Crystals were grown for one week at 20°C and frozen in a cryoprotectant consisting of 0.1 M MES pH 6.5 and 2.25 M MgSO_4_. The Se-Met crystals were produced with the same conditions as for the wild-type protein.

Both the wild-type data and single anomalous dispersion data were collected at station of the Shanghai Synchrotron Radiation Facility (SSRF). The latter was collected at the peak wavelength for Se atom, and then processed using the software HKL2000 [38]. The wild-type crystals of CARD protein gave the best diffraction to 1.75 Å with the space group ***P***4_1_32 and unit cell dimensions of ***a*** = ***b*** = ***c*** = 82.285 Å. The Se-Met substituted crystals were diffracted to 2.5 Å with the same space group and unit cell dimensions ***a*** = ***b*** = ***c*** = 82.370 Å.

### Structure determination and refinement

The program HKL2MAP [39] was used to search for two Se sites in one asymmetric unit and the initial SAD phases were calculated by PHENIX software [40]. The initial molecular model was built automatically by the PHENIX program package and then refined manually by COOT [41] program on the basis of 2***F***o-***F***c and ***F***o-***F***c difference Fourier maps. The structure model was refined using PHENIX program and COOT program iteratively. The final structure had an ***R***_cryst_ value of 19.2% and an ***R***_free_ value of 23.2%. The Ramachandran plot calculated by the program PROCHECK [42] showed that 99% of the residues were in their most favored regions, 1% of the residues were in additionally allowed regions and none of the residues were in generously allowed regions. Detailed data collection and refinement statistics were summarized in Table 1.

**Table 1:** Data collection and refinement statistics.

Crystal name	Wild-type	Se-Met-crystal
Space group	*P*4_1_32	*P*4_1_32
Unit cell (Å)	***a*** = ***b*** = ***c*** = 82.285	***a*** = ***b*** = ***c*** = 82.370
Wavelength (Å)	1.5418	0.9795
Resolution range (Å)	50–1.75 (1.81-1.75)^a^	50–2.52 (2.52-2.37)^a^
No. of unique reflections	9,648	6,549
Redundancy	9.4(2.8)^a^	38.7(39.8)^a^
***R***_sym_ (%)^b^	4.1(40)^a^	8.6(14.2)^a^
***I***/***σ***	46.1(2.6)^a^	57.8(37.8)^a^
Completeness (%)	95.2(78.1)^a^	99.9(100)^a^
FOM		0.763
Refinement		
Resolution range (Å)	21.99∼1.75	
***R***_crystal_ (%)^c^	19.2	
***R***_free_ (%)^d^	23.2	
RMSD_bond_ (Å)	0.007	
RMSD_angle_(°)	1.014	
Number of		
Protein atoms	739	
Ligand atoms	20	
Solvent atoms	89	
Residues in (%)		
most favored	75	
additional allowed	4	
Generously allowed	0	
disallowed	0	
Average B factor (Å^2^) of		
Protein	20.48	
Ligand atoms	37.40	

a the highest resolution shell.

b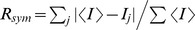.

c ***R***_crystal_ = 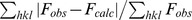.

d ***R***_free_, calculated the same as ***R***_crystal_, but from a test set containing 5% of data excluded from the refinement calculation.

### Cell culture and plasmid construction

HEK293T cells were cultured in Dulbecco's Modified Eagle's Medium (DMEM; Sigma-Aldrich, Co.) supplemented with 10% v/v fetal bovine serum (FBS, HyClone; Thermo Fisher Scientific, Inc.). The cells were maintained in a 95% air/5% CO_2_ environment at 37°C. Mouse CARMA1 encoding full-length protein or only the CARD domain of CARMA1 (residues 1–151) was cloned into the pCMV-Myc vector (Clontech, Co.). Full length or the CARD domain of mouse BCL10 (residues 1–121) was cloned into the pEGFP-C1 vector (Clontech, Co.). The CARMA1 (R35A, K41A, K69A and R72A) and BCL10 (E50A, E53A and E54A) mutants were generated using standard PCR-based mutagenesis methods and confirmed by DNA sequencing.

### Co-immunoprecipitation and western blotting

Expression plasmids GFP-BCL10 (1–121) and Myc-CARMA1 (1–155) were co-transfected into HEK293T cells using PEI (Sigma). Cells were lysed in buffer D containing 20 mM Tris-HCl pH 7.5, 150 mM NaCl, 10% glycerol, 0.5% NP-40 and a Roche protease inhibitor cocktail and were centrifuged at 16,000 *g* for one hour. Thirty microliters of protein A/G agarose (Pierce) was incubated with 1 µg anti-c-Myc antibody in buffer D for two hours in advance and was added to the preformed cell extract and incubated for four hours at 4°C. The beads were washed three times with lysis buffer, and 20 µl of SDS-loading buffer was added. The samples were subjected to SDS-PAGE and western blot analysis.

### Transient transfection and luciferase reporter assay

Full-length CARMA1 and BCL10 were used in the NF-κB reporter assay. HEK293T cells were cultured in 96-well plates and transfected using Lipofectamine 2000 (Invitrogen, Inc.) with 0.1 µg of pGL4.32 [luc2P/NF-κB-RE/Hygro] plasmid (Firefly luciferase reporter plasmid, Promega), 0.06 µg of pGL4.74 [hRluc/TK] vector (Renilla luciferase reporter plasmid, Promega) and 0.01 µg CARMA1 (pCMV-Myc) or BCL10 (pEGFP-C_1_) variants. Twenty hours later, the cell lysates were harvested, and luciferase activities were measured using a Dual-Glo® Luciferase assay kit (Promega). Firefly luciferase expression levels were normalized to the Renilla luciferase transfection control; pCMV-Myc or pEGFP-C_1_ plasmids served as the negative controls. Values shown are averages from representative experiments, and each transfection was performed in duplicate. Meantime, the cell lysates of wild type and mutants were boiled in SDS sample loading buffer, separated by SDS-PAGE and analyzed using immunoblotting with antibodies.

## Supporting Information

Table S1 **Raw data of Luciferase assay.**
(DOC)
