# Supplementary material for: Structural Insights into the Assembly of CARMA1 and BCL10
Source: PLoS One. 2012 Aug 3;7(8):e42775. doi: 10.1371/journal.pone.0042775 (PMC3411838; doi:10.1371/journal.pone.0042775)
Supplement: Table S1 — Raw data of Luciferase assay. (DOC) [file pone.0042775.s001.doc]

Table S1 Raw data of Luciferase assay

| Reporter assay (Wild type and mutants of Carma1) | | | | | | | | | |
| --- | --- | --- | --- | --- | --- | --- | --- | --- | --- |
| Firefly Luciferase Activity | | | | | | | | | |
| Blank | | Control | WT | | | R35A | K41A | K69A | R72A |
| 854 | | 302503 | 1698237 | | | 395662 | 433707 | 473404 | 222716 |
| 542 | | 305910 | 2494045 | | | 225918 | 93445 | 309405 | 231622 |
| 776 | | 277380 | 1684990 | | | 140426 | 62730 | 149971 | 109601 |
| 48 | | 5443 | 39260 | | | 8300 | 8140 | 8806 | 8286 |
| 62 | | 5385 | 11403 | | | 6757 | 7365 | 5745 | 6065 |
| 44 | | 4094 | 63577 | | | 5421 | 10197 | 4709 | 4174 |
| 326 | | 73412 | 422079 | | | 71287 | 38675 | 60814 | 58329 |
| 322 | | 23896 | 248839 | | | 20723 | 37148 | 28530 | 19227 |
| 268 | | 23025 | 243492 | | | 20144 | 36365 | 28054 | 18366 |
| Renilla Luciferase Activity | | | | | | | | | |
| Blank | Control | | | WT | R35A | | K41A | K69A | R72A |
| 798 | 326329 | | | 65296 | 569516 | | 554632 | 475528 | 270862 |
| 508 | 358882 | | | 78319 | 330638 | | 112358 | 333556 | 309135 |
| 448 | 361899 | | | 61675 | 262749 | | 77891 | 204075 | 152923 |
| 38 | 7525 | | | 2662 | 7932 | | 8120 | 7822 | 6831 |
| 46 | 5313 | | | 2414 | 4454 | | 3676 | 8524 | 6305 |
| 32 | 4294 | | | 6725 | 8460 | | 10053 | 5363 | 6271 |
| 262 | 134216 | | | 88969 | 146391 | | 100777 | 102745 | 142068 |
| 190 | 40314 | | | 36860 | 46961 | | 40607 | 46901 | 50245 |
| 222 | 50034 | | | 30427 | 31006 | | 41767 | 53822 | 36477 |

| Reporter assay (Wild type and mutants of Bcl10) | | | | | |
| --- | --- | --- | --- | --- | --- |
| Firefly Luciferase Activity | | | | | |
| Blank | Control | WT | E50A | E53A | E54A |
| 74 | 9388 | 90880 | 24303 | 14860 | 16737 |
| 76 | 4160 | 49834 | 17934 | 8352 | 17722 |
| 52 | 4899 | 25430 | 11361 | 4120 | 2318 |
| 86 | 31501 | 337343 | 88196 | 36233 | 65334 |
| 245 | 25686 | 240587 | 85395 | 37016 | 61015 |
| 289 | 27585 | 282306 | 74516 | 38413 | 44147 |
| 234 | 27214 | 170482 | 54013 | 46127 | 45732 |
| 320 | 39741 | 293937 | 126363 | 40002 | 59438 |
| 276 | 36225 | 367359 | 108394 | 45850 | 41467 |
| 189 | 19235 | 292553 | 111246 | 51544 | 66783 |
| Renilla Luciferase Activity | | | | | |
| Blank | Control | WT | E50A | E53A | E54A |
| 34 | 5125 | 5573 | 4146 | 8906 | 6003 |
| 42 | 2730 | 3104 | 2940 | 3240 | 7147 |
| 26 | 3580 | 1972 | 2316 | 2880 | 2076 |
| 54 | 30289 | 33642 | 27214 | 43243 | 37501 |
| 188 | 23121 | 24791 | 30593 | 28090 | 26946 |
| 158 | 28863 | 21151 | 22236 | 16193 | 58409 |
| 162 | 21323 | 17822 | 18292 | 18623 | 56744 |
| 210 | 55968 | 46460 | 47821 | 51326 | 43046 |
| 220 | 45027 | 49136 | 35654 | 39246 | 88736 |
| 176 | 31441 | 30243 | 29732 | 25064 | 101941 |
